# Differential Regulation of Thermodynamic Binding Forces of Levocetirizine and (*S*)-Cetirizine by Lys191 in Human Histamine H_1_ Receptors

**DOI:** 10.3390/ijms19124067

**Published:** 2018-12-15

**Authors:** Shigeru Hishinuma, Yuri Tamura, Chihiro Kobayashi, Chizuru Akatsu, Masaru Shoji

**Affiliations:** Department of Pharmacodynamics, Meiji Pharmaceutical University, 2-522-1 Noshio, Kiyose, Tokyo 204-8588, Japan; r188902@std.my-pharm.ac.jp (Y.T.); chibi.1206.koba@gmail.com (C.K.); akatsu.imm@mri.tmd.ac.jp (C.A.); msji@my-pharm.ac.jp (M.S.)

**Keywords:** affinity, enthalpy, entropy, histamine H_1_ receptor, levocetirizine, (*S*)-cetirizine

## Abstract

Cetirizine is a zwitterionic second-generation antihistamine containing *R*- and *S*-enantiomers, levocetirizine, and (*S*)-cetirizine. Levocetirizine is known to have a higher affinity for the histamine H_1_ receptors than (*S*)-cetirizine; ligand-receptor docking simulations have suggested the importance of the formation of a salt bridge (electrostatic interaction) between the carboxylic group of levocetirizine and the Lys191 residue at the fifth transmembrane domain of human histamine H_1_ receptors. In this study, we evaluated the roles of Lys191 in the regulation of the thermodynamic binding forces of levocetirizine in comparison with (*S*)-cetirizine. The binding enthalpy and entropy of these compounds were estimated from the van ‘t Hoff equation, by using the dissociation constants obtained from their displacement curves against the binding of [^3^H]mepyramine to the membrane preparations of Chinese hamster ovary cells expressing wild-type human H_1_ receptors and their Lys191 mutants to alanine at various temperatures. We found that the higher binding affinity of wild-type H_1_ receptors for levocetirizine than (*S*)-cetirizine was achieved by stronger forces of entropy-dependent hydrophobic binding of levocetirizine. The mutation of Lys191 to alanine reduced the affinities for levocetirizine and (*S*)-cetirizine, through a reduction in the entropy-dependent hydrophobic binding forces of levocetirizine and the enthalpy-dependent electrostatic binding forces of (*S*)-cetirizine. These results suggested that Lys191 differentially regulates the binding enthalpy and entropy of these enantiomers, and that Lys191 negatively regulates the enthalpy-dependent electrostatic binding forces of levocetirizine, contrary to the predictions derived from the ligand-receptor docking simulations.

## 1. Introduction

Histamine H_1_ receptors, which belong to the family of seven transmembrane receptors, coupled with G_q/11_ proteins, are involved in a variety of physiological and pathophysiological conditions, including allergy and inflammation in peripheral tissues, and the state of arousal in the central nervous system [1,2,3,4,5]. Accordingly, various antihistamines (antagonists or inverse agonists against histamine H_1_ receptors) have been developed for the treatment of type I hypersensitivity, such as allergic rhinitis [6,7,8,9]. Cetirizine is a zwitterionic second-generation antihistamine that has fewer side effects than those caused by first-generation antihistamines, such as sedation, hypnosis, and cognitive impairment, which result from the blockade of H_1_ receptors in the central nervous system [10,11,12]. Cetirizine has the following optical isomers: levocetirizine, the *R*-enantiomer, and (*S*)-cetirizine (the *S*-enantiomer). Levocetirizine has a higher affinity for H_1_ receptors than (*S*)-cetirizine [11,13]; it has been suggested that levocetirizine has a slower dissociation rate than (*S*)-cetirizine at the H_1_ receptors, owing to the interaction with the Lys191 residue in the fifth transmembrane domain of the human histamine H_1_ receptors [13]. Ligand-receptor docking simulations performed based on the crystal structure of the human histamine H_1_ receptor complex with doxepin indicated that the carboxylic group of levocetirizine appeared to form a salt bridge with the Lys191 residue [14]. It was predicted, therefore, that the electrostatic interaction of levocetirizine with Lys191 may be more important for the determination of its binding affinity for H_1_ receptors than that of (*S*)-cetirizine.

Thermodynamic analyses are useful methods for the evaluation of the electrostatic and hydrophobic binding forces of ligands to determine their binding affinity for receptors [15,16,17,18]. Binding enthalpy (∆*H*º) is usually associated with electrostatic binding forces via the formation of new bonds between receptors and ligands, such as hydrogen bonds and van der Waals interactions, whereas binding entropy (∆*S*º) is usually characterized as hydrophobic binding forces via the displacement of ordered water molecules coupled to the formation of new hydrophobic interactions. Therefore, it is of interest to examine, through the mutation of Lys191 to alanine in the H_1_ receptors, how Lys191 contributes to the thermodynamic binding forces of levocetirizine and (*S*)-cetirizine. Herein, we present our novel findings, which show that Lys191 differentially regulates the binding enthalpy and entropy of these enantiomers, and that Lys191 negatively regulates the enthalpy-dependent electrostatic binding forces of levocetirizine, contrary to the predictions derived from the ligand-receptor docking simulations.

## 2. Results and Discussion

### 2.1. Binding Affinities of Levocetirizine and (S)-Cetirizine for Hemagglutinin (HA)-Tagged Wild-Type H_1_ Receptor (HA-WT) and HA-Tagged-Lys191 Mutant of H_1_ Receptor to Alanine (HA-K191A)

**Figure 1:**
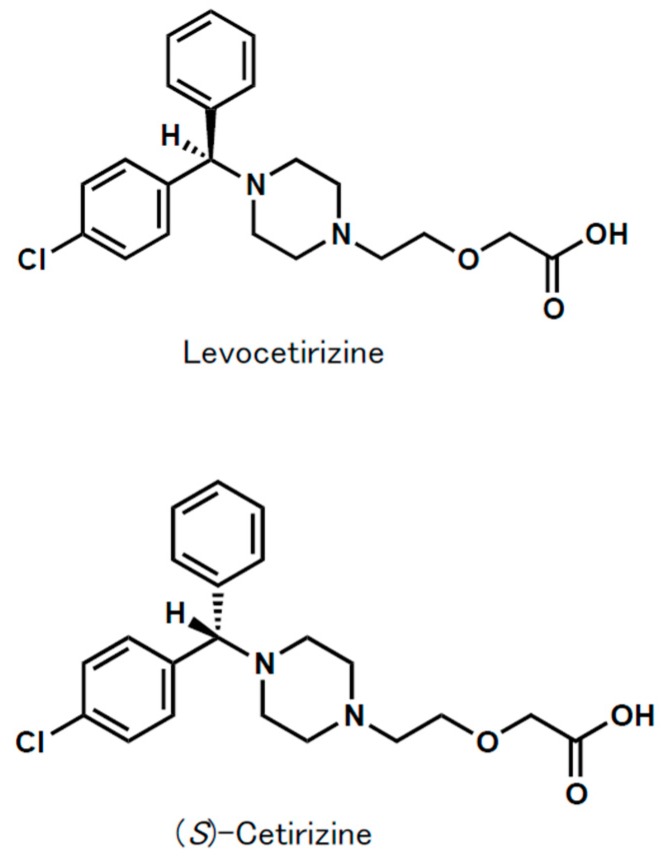
Chemical structures of levocetirizine and (*S*)-cetirizine.

**Figure 2:**
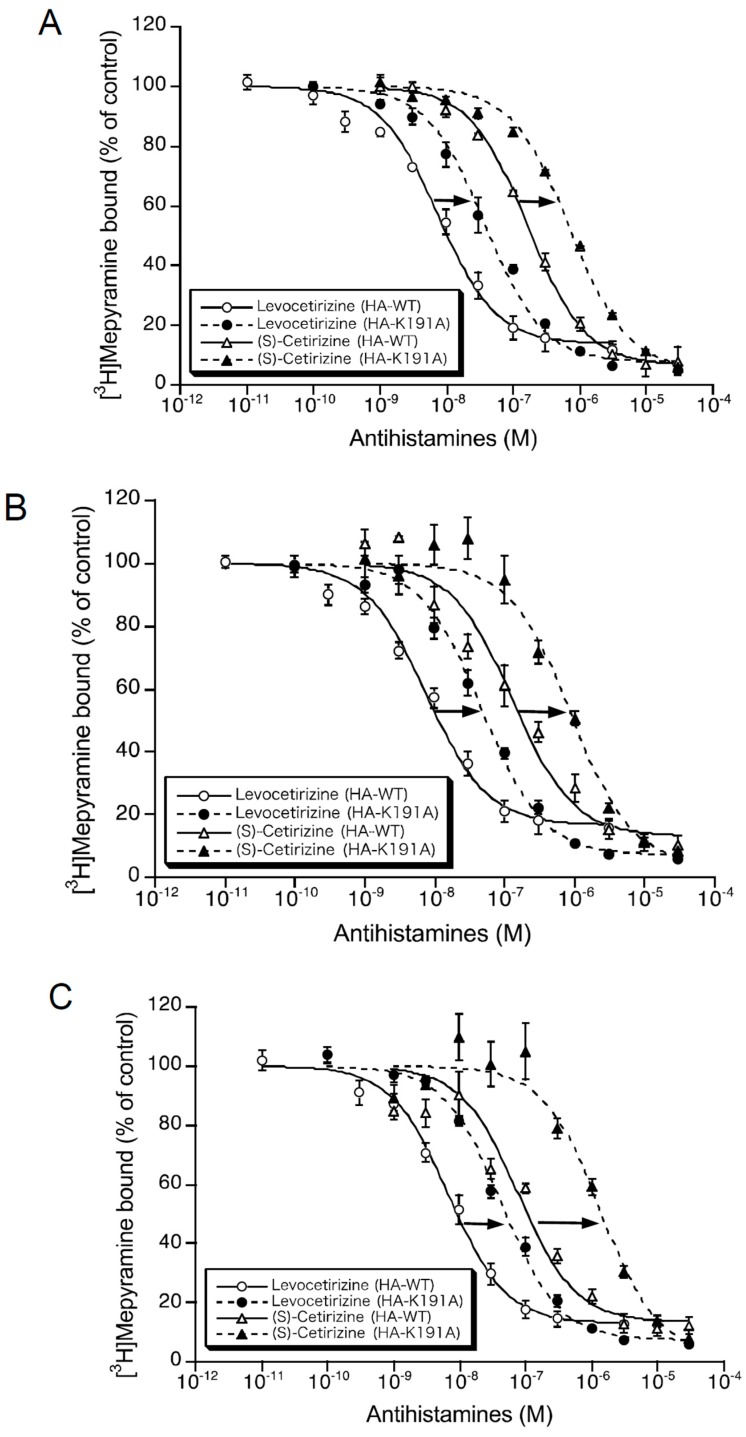
Displacement curves for levocetirizine and (*S*)-cetirizine against the binding of [^3^H]mepyramine to hemagglutinin (HA)-tagged wild-type H_1_ receptor (HA-WT) and HA-tagged-Lys191 mutant of H_1_ receptor to alanine (HA-K191A). The binding of the 3 nM [^3^H]mepyramine to membranes containing HA-WT (open symbols) and HA-K191A (closed symbols), in the presence or absence of various concentrations of levocetirizine (circles) and (*S*)-cetirizine (triangles), was measured at 37 (**A**), 25 (**B**), and 14 °C (**C**), as described in Materials and Methods. The data points are the percentages of bound [^3^H]mepyramine, with 100% as 3 nM [^3^H]mepyramine binding in the absence of displacers, and are the mean ± SEM of three–five independent experiments determined in quadruplicate. The lines are the best-fit curves to a one-site model. Arrows indicate changes induced by the mutation of Lys191 to alanine.

The chemical structures of levocetirizine and (*S*)-cetirizine are shown in Figure 1. To evaluate the binding affinities of levocetirizine and (*S*)-cetirizine for human H_1_ receptors, the inhibitory concentration (IC)_50_ values for these compounds were first obtained from the displacement curves of the binding of 3 nM [^3^H]mepyramine to the membrane preparations of Chinese hamster ovary (CHO) cells expressing HA-WT and HA-K191A at 37 (Figure 2A), 25 (Figure 2B), and 14 °C (Figure 2C). The *K*_i_ values for these compounds were then calculated from the IC_50_ values, as described in Materials and Methods.

**Figure 3:**
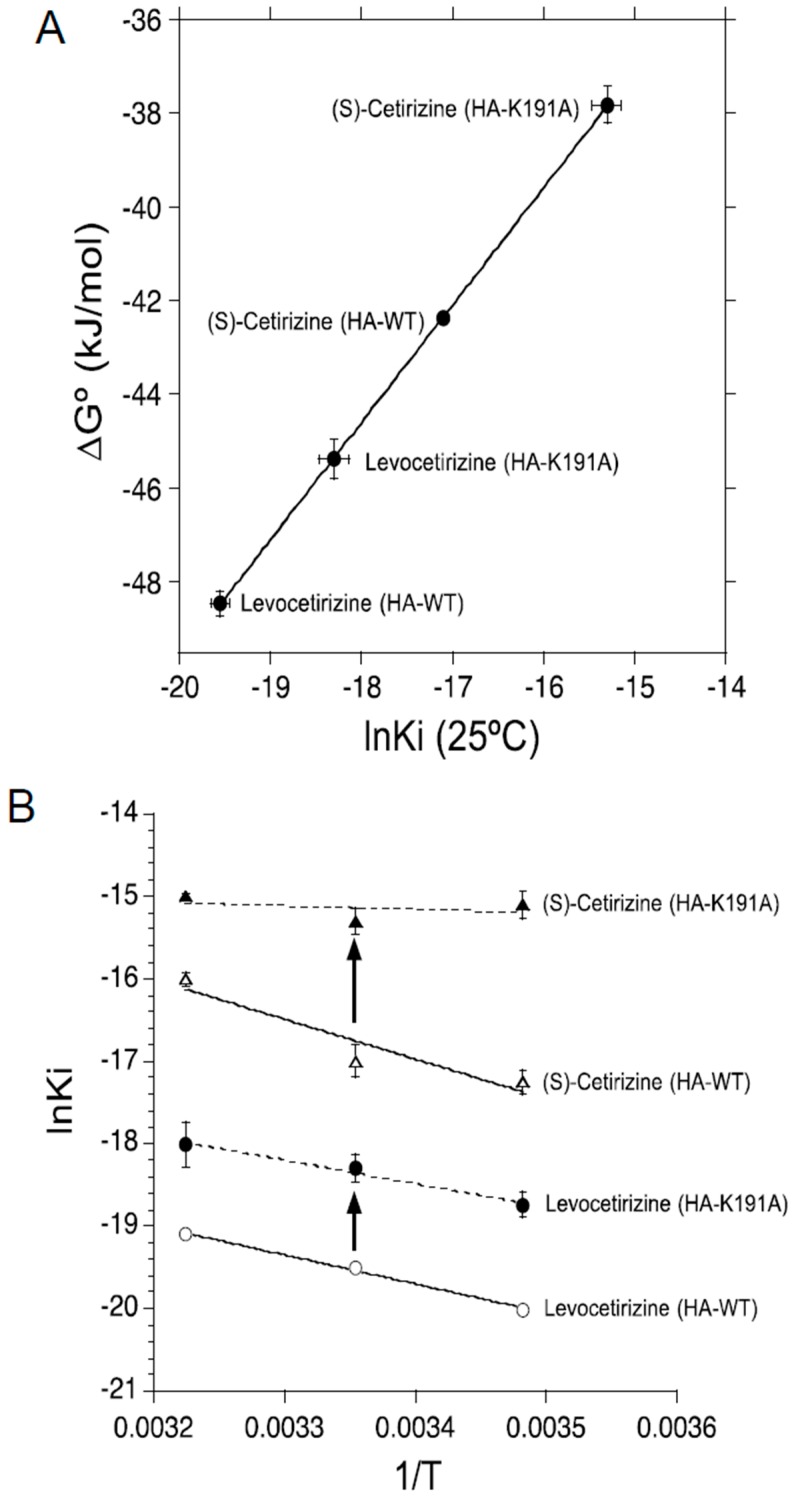

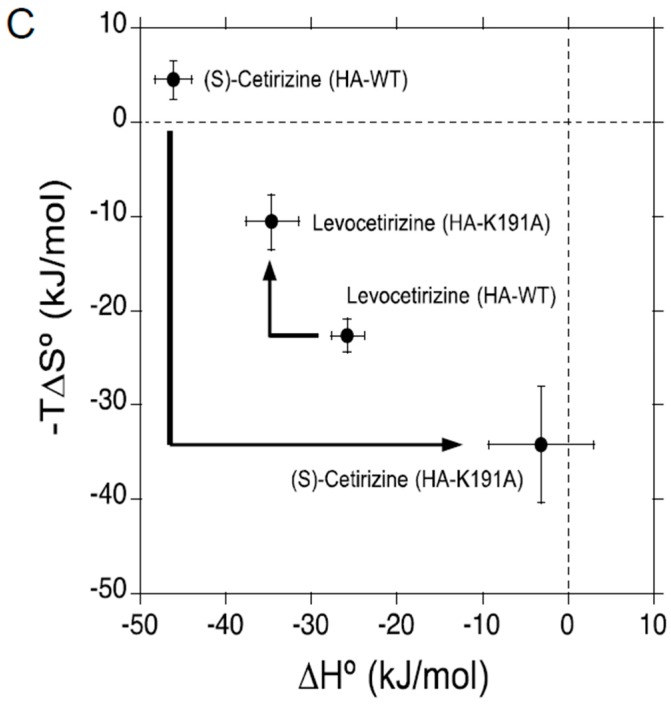
Thermodynamic binding properties of levocetirizine and (*S*)-cetirizine. (**A**) Scatter plots of values of ∆*G*º versus ln*K*_i_ at 25 °C: increases in ∆*G*º and ln*K*_i_ represent reductions in the affinities of ligands for H_1_ receptors; (**B**) van ‘t Hoff plots for levocetirizine and (*S*)-cetirizine: According to the van ‘t Hoff equation, ln*K*_i_ = ∆*H*º/*RT* − ∆*S*º/*R*, the slope and intercept of the vertical axis represent ∆*H*º/*R* and −∆*S*º/*R*, respectively; (**C**) Scatter plots of values of −*T*∆*S*º versus ∆*H*º: compounds with a negative value of ∆*H*º and positive value of −*T*∆*S*º are classified as enthalpy-driven; conversely, compounds with a positive value of ∆*H*º and negative value of −*T*∆*S*º are classified as entropy-driven. Compounds with negative values of ∆*H*º and −*T*∆*S*º are classified as enthalpy- and entropy-driven. Reductions in the values of ∆*H*º and −*T*∆*S*º represent increases in the binding forces of the ligands mediated via enthalpy and entropy, respectively. Arrows indicate changes induced by the mutation of Lys191 to alanine.

The ln*K*_i_ values for levocetirizine and (*S*)-cetirizine and their corresponding ∆*G*º values (∆*G*º = *RT*ln*K*_i_) at a standard temperature of 25 °C (298.15 K) are shown in Figure 3A. The *K*_i_ values of levocetirizine and (*S*)-cetirizine for HA-WT were 3.31 ± 0.45 nM (*n* = 4) and 39.1 ± 7.00 nM (*n* = 5), respectively (Figure 3A; HA-WT). Thus, the affinity of levocetirizine for HA-WT was approximately 12 times higher than that of (*S*)-cetirizine (*p* < 0.001). These results were in good accordance with a previous report showing the higher affinity for levocetirizine than (*S*)-cetirizine [11].

By the mutation of Lys191 to alanine, the *K*_i_ values for levocetirizine and (*S*)-cetirizine were significantly increased to 11.9 ± 1.80 nM (*n* = 3) and 244 ± 34 nM (*n* = 4), respectively (Figure 3A; HA-K191A). Thus, the affinity for levocetirizine and (*S*)-cetirizine were reduced approximately 3.6 and 6.2 times, respectively, by this mutation. These results were also in good agreement with a previous report showing a significant role of Lys191 in the regulation of the affinity for these compounds [13].

### 2.2. Thermodynamic Binding Forces of Levocetirizine and (S)-Cetirizine to HA-WT and HA-K191A

To evaluate the thermodynamic binding forces of levocetirizine and (*S*)-cetirizine that are responsible for the higher affinity of levocetirizine than (*S*)-cetirizine, as well as for the reduction in their affinity for H_1_ receptors caused by the mutation of Lys191 to alanine, van ‘t Hoff plots were constructed in order to estimate their thermodynamic binding forces, that is, the binding enthalpy (∆*H*º) and entropy (-*T*∆*S*º) of levocetirizine and (*S*)-cetirizine to HA-WT and HA-K191A from the slope-intercept equation, ln*K*_i_ = ∆*H*º/*RT* – ∆*S*º/*R* (Figure 3B).

Figure 3C shows the scatter plots of values of -*T*∆*S*º versus ∆*H*º for these compounds, obtained from van ‘t Hoff plots (Figure 3B). In the binding of levocetirizine and (*S*)-cetirizine to HA-WT (Figure 3C; HA-WT), negative values of ∆*G*º (= ∆*H*º – *T*∆*S*º) for (*S*)-cetirizine were predominantly achieved by enthalpy (∆*H*º), whereas those for levocetirizine were caused by both enthalpy (∆*H*º) and entropy (-*T*∆*S*º). Thus, (*S*)-cetirizine appeared to bind to the H_1_ receptors predominantly via the enthalpy-dependent electrostatic binding forces, whereas levocetirizine appeared to bind to the H_1_ receptors via the entropy-dependent hydrophobic binding forces, in addition to the enthalpy-dependent electrostatic binding forces. It is most likely, therefore, that the higher affinity for levocetirizine than (*S*)-cetirizine, as well as the slower dissociation rate of levocetirizine than (*S*)-cetirizine [13], are caused by the larger entropy-dependent hydrophobic binding forces of levocetirizine than (*S*)-cetirizine.

By the mutation of Lys191 to alanine, the entropy-dependent hydrophobic binding forces of levocetirizine were significantly reduced (Figure 3C; HA-K191A), which appears to explain the reduction in the affinity of levocetirizine by this mutation. Interestingly, the enthalpy-dependent electrostatic binding forces of levocetirizine were significantly increased by this mutation. Thus, Lys191 appeared to play an inhibitory role in the electrostatic binding forces of levocetirizine. These results were contrary to the predictions derived from the ligand-receptor docking simulations, in that the electrostatic interaction of levocetirizine with Lys191 might play a crucial role in maintaining its binding affinity for H_1_ receptors [14]. On the other hand, the enthalpy-dependent binding forces of (*S*)-cetirizine were significantly reduced by the mutation of Lys191 to alanine (Figure 3C; HA-K191A), which appeared to explain the reduction in the affinity of (*S*)-cetirizine by this mutation. Thus, it was revealed that the enthalpy-dependent electrostatic interaction of Lys191 was more important for the binding of (*S*)-cetirizine than levocetirizine.

In conclusion, the entropy-dependent hydrophobic interaction was revealed to be the cause of the higher affinity of levocetirizine compared with (*S*)-cetirizine. Furthermore, Lys191 was revealed to differentially regulate the thermodynamic binding forces of levocetirizine and (*S*)-cetirizine, so as to determine the affinity of these enantiomers for H_1_ receptors (summarized in Table 1). These findings have provided novel insight into the mechanisms by which the affinity of enantiomers for their receptors are differentially determined by their thermodynamic binding forces.

## 3. Materials and Methods

### 3.1. Materials

[Pyridinyl-5-^3^H]-mepyramine was purchased from PerkinElmer (Waltham, MA, USA). Levocetirizine dihydrochloride was purchased from LKT Laboratories (St. Paul, MN, USA), and (*S*)-cetirizine dihydrochloride was purchased from Toronto Research Chemicals (Toronto, Ontario, Canada). Chinese hamster ovary cells (CHO-K1: RCB0285, RRID: CVCL_0214) were purchased from the RIKEN Bioresource Center (Tsukuba, Ibaraki, Japan). The expression vectors (3× HA hH1R/pcDNA3.1(+)) for the wild-type human histamine H_1_ receptors tagged with three molecules of hemagglutinin (HA: YPYDVPDYA) at the N-terminus were purchased from the Missouri S&T cDNA Resource Center (Rolla, MO, USA). Other materials were purchased from Sigma-Aldrich (Tokyo, Japan).

### 3.2. Preparation of Cells and Membrane Fraction

The experimental protocols of this research were approved by the Institutional Safety Committee for Recombinant DNA Experiments, Meiji Pharmaceutical University (no. 1209). The study was not pre-registered and required neither randomization nor blinding. The construction of CHO cells stably expressing HA-tagged wild-type human histamine H_1_ receptors (HA-WT) has been described previously [19]. The expression vectors for the Lys191 mutants of HA-WT to alanine (HA-K191A) were constructed using the PrimeSTAR mutagenesis basal kit (Takara Bio, Otsu, Shiga, Japan) and Mastercycler Gradient (Eppendorf, Hamburg, Germany) in 3 × HA hH1R/pcDNA3.1(+), in accordance with the manufacturer’s protocol [20,21]. The nucleotide sequences of the mutated H_1_ receptor genes were confirmed by using an ABI PRISM Genetic Analyzer 310A with ABI PRISM BigDye Terminator ver. 3.0 (Applied Biosystems, Tokyo, Japan). The cells expressing HA-WT and HA-K191A were cultured in 150 cm^2^ culture flasks, as described previously [18]. The dissociated cells were homogenized with Polytron PT-10 (Kinematica, Lucerne, Switzerland) in an ice-cold lysis buffer (Tris, 1 mM; EDTA, 2 mM; pH 7.4 at 37 °C). The homogenate was centrifuged at 1000 *g* for 10 min at 4 °C, and the supernatant was centrifuged at 100,000 *g* for 60 min at 4 °C. The pellet was suspended in an ice-cold normal HEPES buffer (NaCl, 120 mM; KCl, 5.4 mM; MgCl_2_, 1.6 mM; CaCl_2_, 1.8 mM, D-glucose, 11 mM; and HEPES, 25 mM; pH 7.4 at 37 °C) for the subsequent binding assay.

### 3.3. Measurement of [^3^H]mepyramine Binding to Membrane Preparations

The receptor binding assay with [^3^H]mepyramine, a radioligand for H_1_ receptors, was performed in accordance with the methods described previously [18], as follows: Aliquots (0.1 mL) of membrane preparations (approximately 50 µg of membrane proteins) were incubated with 3 nM [^3^H]mepyramine in the presence or absence of various concentrations of levocetirizine or (*S*)-cetirizine (displacement experiments) for 3 h at 37 °C, 24 h at 25 °C, and 3 days at 14 °C in a normal HEPES buffer (final volume 1 mL). The actual concentration of [^3^H]mepyramine present was determined by the analysis of an aliquot of the [^3^H]mepyramine/HEPES medium. The reaction mixture was filtered through Whatman GF/B glass fiber filters (pre-soaked for at least 3 h in 0.3% polyethylenimine), using a 24-place cell harvester (Brandel, Gaithersburg, MD, USA). The radioactivity trapped on the filters was determined by scintillation counting. All of the determinations were made in quadruplicate. The protein content of the membrane preparations was determined using a bicinchoninic acid (BCA) protein assay kit (Pierce, Rockford, IL, USA).

### 3.4. Data Analyses

All of the data were presented as the mean ± SEM of at least three measurements performed in quadruplicate. The statistical significance was evaluated by the Student’s *t*-test or an ANOVA with the Bonferroni correction. A value of *p* < 0.05 was considered significant.

The IC_50_ values for displacers (levocetirizine and (*S*)-cetirizine) were determined by fitting the displacement curves to the one-site model (KaleidaGraph, version 4.1, Synergy Software, Reading, PA, USA), as follows:

*B* = 100 − *P* × *C*/(*C* + *IC*_50_) (1)

where *B* is the amount of [^3^H]mepyramine bound, *C* is the concentration of the displacer, and *P* and IC_50_ are the percentages of the binding sites and IC_50_ values for the displacer, respectively.

The *K*_i_ values for the displacers were then estimated from the Cheng and Prusoff equation, as follows [18,22]:

*K*_i_ = *IC*_50_/(*C*/*K*_d_ + 1)(2)

where *K*_i_ and IC_50_ are the dissociation constant and IC_50_ value for the displacer, respectively, *C* is the concentration of [^3^H]mepyramine, and *K*_d_ is the dissociation constant for [^3^H]mepyramine at each temperature.

The thermodynamic binding parameters for the ligands (levocetirizine and (*S*)-cetirizine) were assessed by the Gibbs and van ‘t Hoff equations, as follows [16,18]:

∆*G*º = *RT*ln*K*_i_ = ∆*H*º − *T*∆*S*º (3)

where ∆*G*º is the standard free energy, *R* is the gas constant (8.314 J/K/mol), *T* is the absolute temperature, *K*_i_ is the dissociation constant for ligands, and ∆*H*º and ∆*S*º are the standard enthalpy and entropy, respectively.

## Figures and Tables

**Table 1:** Changes in the binding parameters for levocetirizine and (*S*)-cetirizine by the mutation of Lys191 to alanine in hemagglutinin (HA)-tagged human H_1_ receptors. * *p* < 0.05, ** *p* < 0.01; the binding affinity and thermodynamic binding forces for levocetirizine and (*S*)-cetirizine were significantly reduced (**↓**) or increased (**↑**) by the mutation of Lys191 to alanine.

Antihistamines	Binding Affinity	Thermodynamic Binding Forces	
		Enthalpy-Dependent Electrostatic Binding Forces	Entropy-Dependent Hydrophobic Binding Forces
Levocetirizine	↓**	↑*	↓*
(*S*)-Cetirizine	↓**	↓**	↑**

## Abbreviations

CHO	Chinese hamster ovary
GPCR	G protein-coupled receptor
HA	Hemagglutinin
K191A	Lys191 mutant of H_1_ receptor to alanine
WT	Wild-type H_1_ receptor
